# Characterization of the complete chloroplast genome of *Hippophae tibetana*

**DOI:** 10.1080/23802359.2019.1710595

**Published:** 2020-01-14

**Authors:** Wu Zhou, Qi Dong, Honglun Wang, Na Hu

**Affiliations:** a State Key Laboratory of Plateau Ecology and Agriculture, Qinghai University, Xining, P. R. China;; b College of Eco-Environmental Engineering, Qinghai University, Xining, P. R. China;; c Key Laboratory of Tibetan Medicine Research, Northwest Institute of Plateau Biology, Chinese Academy of Sciences, Xining, P. R. China;; d Qinghai Provincial Key Laboratory of Tibetan Medicine Research, Xining, P. R. China

**Keywords:** *Hippophae tibetana*, chloroplast genome, phylogenetic analysis

## Abstract

*Hippophae tibetana* adapted well to the unique plateau climate, diffused in the Qinghai–Tibetan Plateau–Himalayas. Here, we characterized the complete chloroplast genome of *Hippophae tibetana*. The chloroplast genome is found to be 155,810 bp in length with 36.67% GC contents. The chloroplast genome sequences encoding 131 genes, including 85 mRNA genes, 38 tRNA genes, and 8 rRNA genes. The phylogenetic trees further revealed that *Hippophae tibetana* is an independent species different from the model species *Hippophae rhamnoides* and chloroplast genome sequence showed a significant variation within and between closely related *Hippophae* genus.

*Hippophae tibetana* are widely distributed in the Qinghai–Tibetan Plateau–Himalayas, belonging to the family Elaeagnaceae of the genus *Hippophae*. It adapted well to the dry climate, low-temperature, hypoxia, and stormy plateau weather.

With regard to the morphology, *Hippophae tibetana* closely resemble the model species *Hippophae rhamnoides* Linn., thus Hooker f. just merged *Hippophae tibetana* into the sea-buckthorn species for a variant species in 1886. At present, *Hippophae tibetana* recognized as an independent species and other 6 species and 11 subspecies of *Hippophae* are identified according to morphological characteristics, geographical distribution, and growth environment. However, the evolutionary relationship among the *Hippophae* intrageneric groups still needs to be further distinguished. Chloroplast genome is adequate for the study of genetic diversity and the relationship between and within related species, as an independent and monolepsis genetic unit.

Therefore, in this study, the whole chloroplast genome of *Hippophae tibetana* was characterized by sequencing it using Illumina HiSeq4000 platform, assembled with SPAdes version 3.9.1 (Bankevich et al. 2012) and annotated with CpGAVAS (Liu et al. 2012). Tree specimens of *Hippophae tibetana* were collected from the Qinghai-Tibet Plateau in China (N37°36′53.34′′, E101°19′18.63′′) and deposited in the Qinghai-Tibetan Plateau Museum of Biology (voucher specimen: zhou2019014).

The phylogenetic analysis was conducted with an alignment of 32 complete chloroplast genome from the order Rosales, with *Arabidopsis thaliana* as the outgroup, using MAFFT (version 7.452) (Katoh et al. 2019) with the FFT-NS-2 strategy. Phylogenies trees were constructed using four methods. such as Bayesian inference (BI) by Mrbayes 3.2.6 (Ronquist and Huelsenbeck 2003), maximum parsimony (MP) by PAUP 4.0, maximum likelihood (ML) by MEGA X (Kumar et al. 2018) and neighbor-joining (NJ) by MAFFT with default parameters (Kuraku et al. 2013). MP was calculated by a heuristic search with 1000 random addition sequence replicates under the Kishino-Hasegawa test. ML analysis preferences were optimized with 1000 bootstrap replications using the Jukes-Cantor model with the Gamma distributed rate among sites. A GTR + F+I + G4 nucleotide substitution model was selected as the best-fit model according to AIC (Kalyaanamoorthy et al. 2017) for the BI analysis.

The length of the complete chloroplast genome (MN643620) of *Hippophae tibetana* is 155,810 bp with 36.67% GC content, encoding 131 genes, including 85 mRNA, 38 tRNA, and 8 rRNA. A total of 246 simple sequence repeats (SSRs) microsatellites were identified and 162 mono-, 10 di-, 69 tri- and 5 tetra-nucleotide repeats were classified. All the protein-coding genes presented a total of 25,339 codons, with leucine (2682 codons, approximately 10.58% of the total codons) as the most abundant amino acid.

Complete sequence cluster analysis of the chloroplast genome revealed that *Hippophae tibetana* formed a single clade and then formed a small branch with the clade established by *Hippophae rhamnoides* (Chen and Zhang 2017) and *Hippophae neurocarpa* (Zhou et al. 2019) for high Bayesian inference and bootstrap values (Figure 1). The phylogenetic trees further revealed that *Hippophae tibetana* is an independent species different from *Hippophae rhamnoides* and chloroplast genome sequence showed a significant variation within and between closely related plant species (Wambugu et al. 2015).

**Figure 1.**
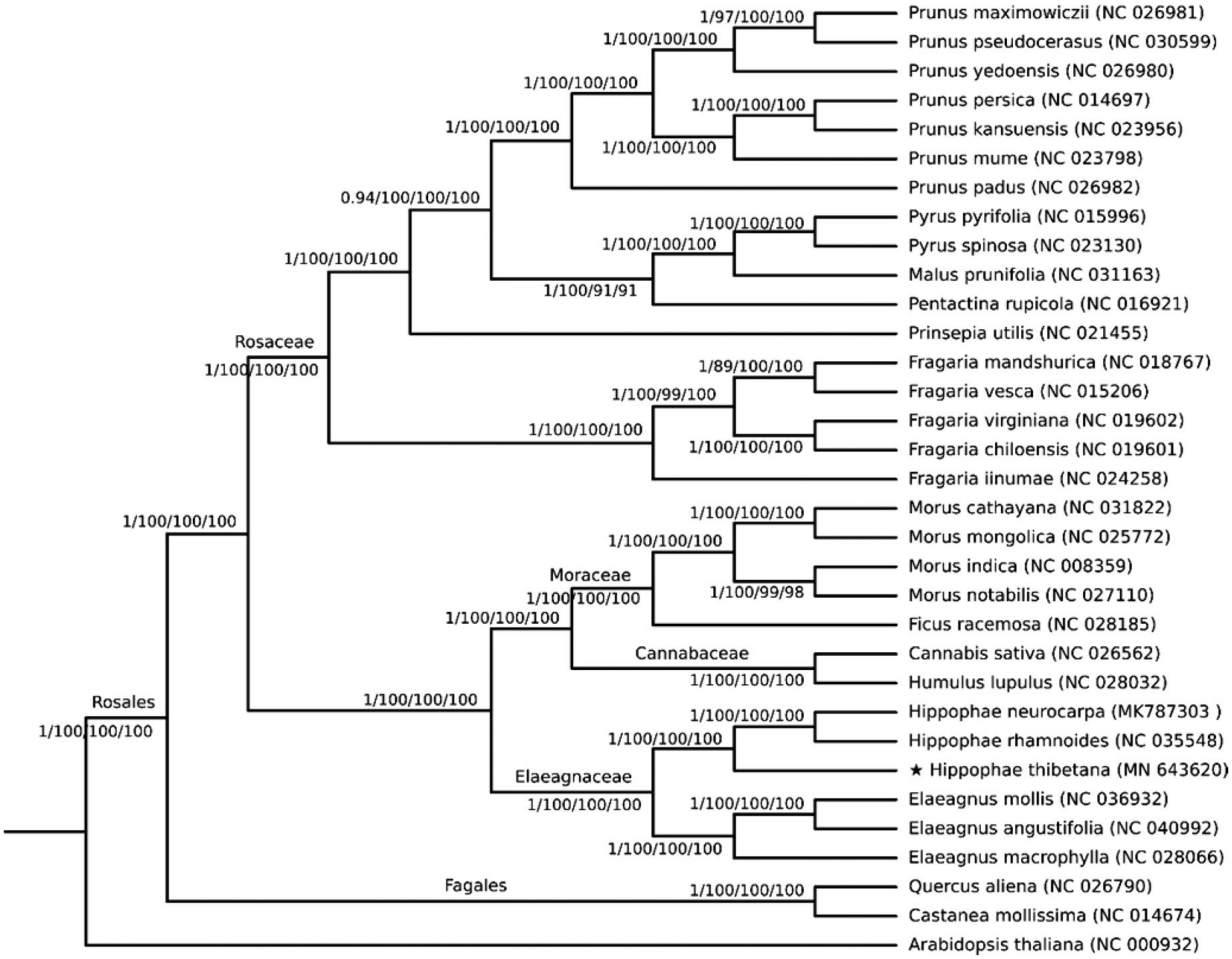
Phylogenetic trees were gathered from 32 complete chloroplast genomes of the order Rosales, with *Arabidopsis thaliana* as the outgroup species, using 4 different methods: Bayesian inference (BI), maximum parsimony (MP), maximum likelihood (ML) and neighbor-joining (NJ). The posterior probability of BI and bootstrap values for MP, ML and NJ are listed above or below the branches. The pentagram indicates the positions of *Hippophae thibetana*.
